# Collaborative HRM, climate for cooperation, and employee intra-organizational social ties in high-technology firms in China: A cross-level analysis

**DOI:** 10.3389/fpsyg.2023.1036113

**Published:** 2023-01-25

**Authors:** Zhongxing Su, Mengfei Zhou, Xiaobei Li, Yuxin Yang, Wei Shi

**Affiliations:** ^1^School of Labor and Human Resources, Renmin University of China, Beijing, China; ^2^Shanghai Business School, Shanghai, China

**Keywords:** collaborative HRM, climate for cooperation, employee social ties, cross-level analysis, high-technology firms

## Abstract

Individual social ties have been an important source of competitive advantages for hightech firms in the knowledge economy. However, the existing cross-level studies have mainly investigated the impact of HRM systems on traditional individual attitudinal or behavioral outcomes, and few studies have examined the effect of SHRM on individual social ties. Based on the data collected from 363 knowledge employees working in 64 high-tech firms in China, we examine the cross-level relationships among collaborative HRM practices, climate for cooperation and employee intra-organizational social ties. The hierarchical linear model results show that organizational-level collaborative HRM practices have significant positive effects on the number and strength of individual-level intra-organizational social ties, and the climate for cooperation mediates the positive cross-level relationship between collaborative HRM and individual intra-organizational social ties. This study makes three contributions to the literature. First, with a cross-level model, this study helps us better understand how collaborative HRM acts as an approach to manage individuals’ social capital formation. Second, this study makes contribution to the social network literature by showing how organizational contextual factors (HRM practices and organizational climate) affect employee individual social ties. Third, based on the AMO model, this paper developed a more clear construct and a three-dimension measurement of the collaborative HRM.

## Introduction

Over the past two decades, scholars have widely recognized the essential role that strategic human resource management (SHRM) plays in driving organizational effectiveness (Huselid, 1995; Combs et al., 2006). Recently, the cross-level effects of the organizational HRM systems (e.g., a bundle of favorable HR practices) on individual employee outcomes have received an increasing amount of attention (Huang et al., 2018; Pahos and Galanaki, 2022). However, the existing cross-level studies have mainly investigated the impact of HRM systems on traditional individual attitude or behavior varialbes, such as job satisfaction, organizational commitment, and organizational citizenship behavior (Kehoe and Wright, 2013; Heffernan and Dundon, 2016; Zhang et al., 2018), and few studies have examined the effect of SHRM on individual social ties.

Individual social tie (or relational tie) is the social links between actors (Hollenbeck and Jamieson, 2015), which is the core of a social network (Seibert et al., 2001). With the advent of the knowledge economy, employee social ties have been an important source of competitive advantages for knowledge intensive companies since the processes of acquiring, transforming and integrating valuable knowledge often occurs in individual social interactions (Nahapiet and Ghoshal, 1998; Kale et al., 2000; Kang et al., 2007). Empirical studies have revealed that social interactions in social networks can influence knowledge exchange and knowledge creation, and ultimately lead to better team and organizational effectiveness (Hansen et al., 2005; Chen and Huang, 2007).

Scholars have highlighted the roles of HRM practices in influencing the way employees interact with their colleagues (Brass et al., 2004; Kaše et al., 2009). For instance, HR practices communicate organization’s rules, procedures, and policies, which are critical in forming employee social ties (e.g., Liebeskind et al., 1996; Leana and Van Buren, 1999). Moreover, HR practices such as extensive training and work design for example provides employees with the structural connections to develop constructive interpersonal interactions, which ultimately lead to firm competitive advantages (Evans and Davis, 2005; Lengnick-Hall and Lengnick-Hall, 2006). Accordingly, several empirical studies have investigated the relationships between the collaboration-based or relation-oriented HRM systems and collective outcomes. For example, Chuang et al. (2016) examined an “HRM system for knowledge-intensive teamwork” and demonstrated its impact on the team-level knowledge generation processes of R&D teams. Kehoe and Collins (2017) reported that a set of relationship-oriented HR practices improved the performance of units by increasing unit members’ aggregate access to knowledge within and outside units. Nieves et al. (2016) reported that collaborative HRM are an antecedent of product innovation. Colakoglu et al. (2021) argued that the perceived collaboration-based HR systems promote information exchange. Chen Y. Y. et al. (2011) found a positive interaction effect of collaborative HR practices reported by employees and network range on objective sales performance. Zhou et al. (2012) proposed that the application of collaboration-based HRM will be positively related to external talent deployment to achieve innovation and accentuate firm performance. However, so far, there is a lack of studies to examine whether and how organizational-level HRM practices will influence individual-level employee social ties.

In this study, we propose that the collaborative HRM, as an HRM configuration investing in interpersonal relationships rather than in individuals (Lepak and Snell, 1999), focusing on permeable and network intimate work structures, team development, and group incentives and emphasizes co-operation, information sharing and knowledge transfer (Lepak and Snell, 1999; Youndt and Snell, 2004), is an important approach to promoting individual social ties. According to the AMO model (Appelbaum et al., 2000; Jiang et al., 2012), we argue that collaborative HRM can directly affect employee social network by enhancing individuals’ ability, motivation and opportunity to build social ties with their colleagues. Furthermore, based on the implication of previous studies that HRM systems usually influence employee outcomes through building a social context (Ferris et al., 1998; Collins and Smith, 2006), we propose that an organizational climate for cooperation will mediate the relationships between collaborative HRM and the number and strength of employees’ intra-organizational social ties.

The number and strength of employees’ social ties are two frequently-used indicators to describe a social network (Senaratne et al., 2021). The number of social ties is usually defined as the number of relationships maintained by individuals in a social network, and it indicates individuals’ influence and power within the social network (Müller-Prothmann, 2007; Borgatti et al., 2013). The strength of social ties describes the degree of connectivity such as the likelihood of information flows among individuals. When the strength is high, individuals are motivated to provide information and support to others (Kim et al., 2011; Borgatti et al., 2013).

**Figure 1:**
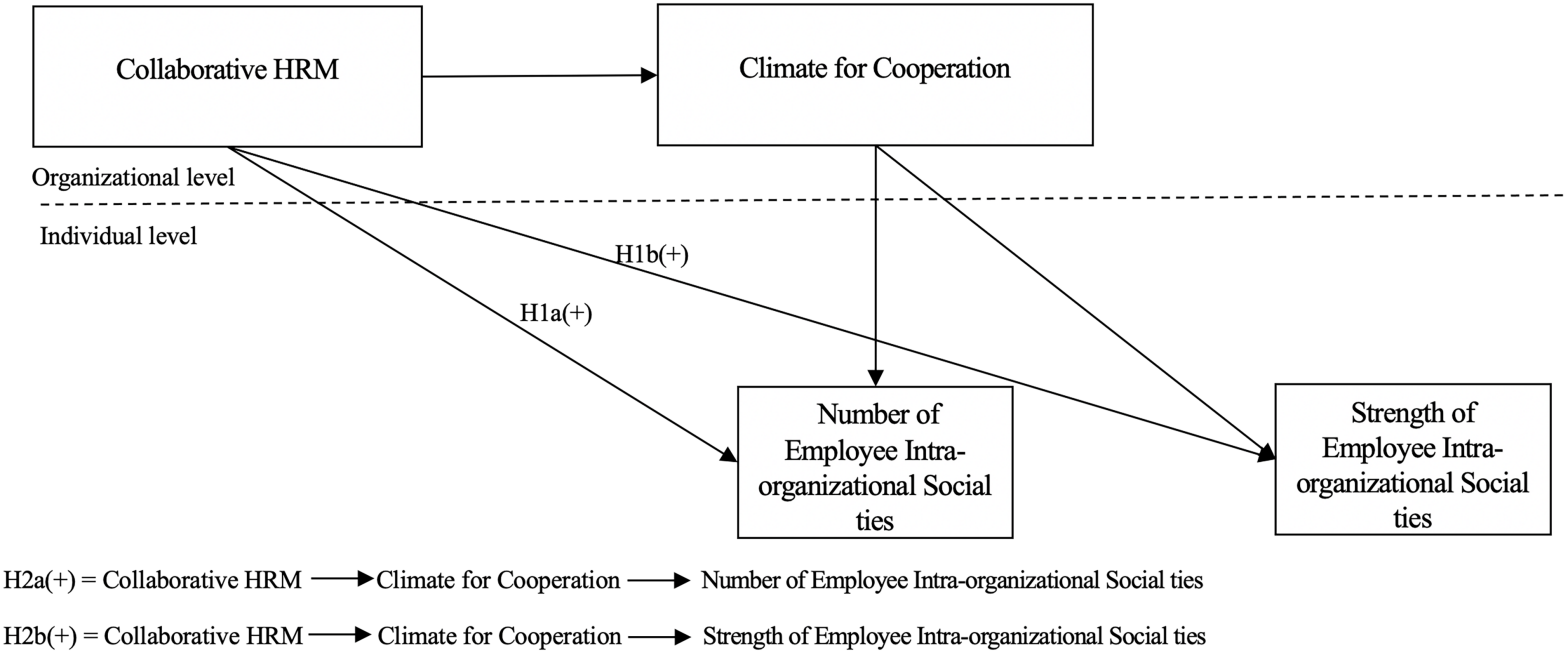
Hypothesized model.

Our research model is shown in Figure 1. As illustrated, we applied the cross-level mediation-upper mediator model, also known as the 2–2-1 model (Zhang et al., 2009). In this model, collaborative HRM and organizational climate for cooperation are variables at the organizational level, and the number and strength of employees’ intra-organizational social ties are variables at the individual level. We collected data from both organizations and employees, and conducted the cross-level analysis with Mplus. We predict that the level-2 independent variable (i.e., collaborative HRM) influences the level-1 dependent variables (i.e., tie number and tie strength) through the level-2 mediating variable (i.e., climate for cooperation).

Our research will make three contributions. First, with a cross-level model, this study helps us better understand how collaborative HRM acts as an approach to manage individuals’ social capital formation. This not only expands the literature on strategically-targeted HRM with a multilevel perspective (Lepak et al., 2006; Takeuchi et al., 2009), but also unfolds the mechanisms that link HRM and employee outcomes. Second, this study makes contribution to the social network literature by showing how organizational contextual factors (HRM practices and organizational climate) affect employee individual social ties. Past research has focused on the individual level antecedents of social ties, such as individuals’ gender, age, and self-monitoring levels (Brass et al., 2004; Osman-Gani and Rockstuhl, 2009), however few studies have investigated organizational antecedents (Kaše et al., 2009). From the SHRM perspective, we demonstrate that organizational HRM practices can be effective tools to develop employee social ties. Third, based on the AMO model, this paper developed a more clear construct and a three-dimension measurement of the collaborative HRM, since previous studies were not so consistent and detailed in the structure and measurement of the collaborative HRM (Lepak and snell, 2002; Lopez-Cabrales et al., 2009).

## Theoretical background and hypotheses

### Collaborative HRM

The pioneer literature studies on collaborative HRM are primarily concerned with information sharing and building cooperative relationship between collaborators, such as enterprises and their suppliers (Lepak and Snell, 1999, 2002). As Lepak and Snell (1999, p. 41) stated, “although alliances may involve structural arrangements in which employees from both parties work together, HR systems that encourage and reward cooperation, collaboration, and information sharing are also likely to be necessary.” Accordingly, they argued that organizations use team-building training, communication mechanisms, and the like to facilitate information sharing and the transfer of knowledge. Later on, the concept of collaborative HRM gained relevance and applicability within organizations. Lopez-Cabrales et al. (2009) argued that collaborative HRM (including teamwork design, teamwork skills-based training, socialization programs, team-based appraisal systems, and collective reward systems) are critical for disseminating specialized knowledge within organizations. Specifically, as a kind of HRM with specific goal orientation, collaborative HRM comprises of a set of HRM practices that promotes interaction and collaboration among employees. For example, Kang et al. (2007) and Lopez-Cabrales et al. (2009) argued that collaborative HRM practices (including teamwork design, job rotation, teamwork skills based selection and training, socialization programs, team-based appraisal systems and collective reward systems) are critical for disseminating specialized knowledge within organizations.

We adopted the widely used ability-motivation-opportunity approach (e.g., see also Combs et al., 2006; Lepak et al., 2006; Jiang et al., 2012) and argue that the collaborative HRM should include ability-enhancing practices, such as employee selection and promotion that prioritize candidates with strong collaborative competency, and workplace mentoring system that binds employees into learning partnership; motivation-enhancing practices such as performance evaluations that gives a substantial weight to employees’ social skills, merit pay increase that is driven by employees’ teamwork skills, and a reward system that actively recognize employees’ performance in collaborative tasks; opportunity-enhancing practices such as rotating jobs, sponsoring employees’ social gatherings, and wide utilization of interdependent work design. Employees who have the ability to cooperate with each other do not necessarily have the motivation to do so, and employees who have both the ability and motivation do not necessarily have the opportunity to cooperate with their colleagues. Consequently, only when all three dimensions are available can social ties be more generated at the individual level.

### Employee intra-organizational social ties

A social network is defined as a relatively stable system of social ties linking a defined set of persons or social actors (Burt, 1997; Tsai and Ghoshal, 1998). Individual social ties are the core of a social network (Seibert et al., 2001). In the organization context, employees’ intra-organizational social ties refer to the social relations that employees have with their colleagues within the organizations, which can be characterized by tie number and strength. More specifically, the tie number conveys how many peers the employee interacts with for information and knowledge exchange, while the tie strength conveys how often and tightly the employee interacts with their colleagues.

Studies have revealed that a greater number of ties help employees obtain a broader range of perspectives (Dean and Brass, 1985), enabling employees to make more extensive use of new information (e.g., policy, technology) introduced in their workplace. In addition, frequent interaction with colleagues helps employees gather information quickly, and close ties with others allow individuals to dispense with formality and self-censorship, and to get to the heart of issues (Pil and Leana, 2009). Besides, since every member of an organization is embedded in a wide and complex social network, when individuals maintain a strong social network (e.g., a social network with high a tie number and tie strength), they are more well connected to the larger social network within the organization, and can be more effective to obtain diversified information (Sosa, 2011; Hirst et al., 2015). Prior studies have shown that strong social interactions or social networks facilitate knowledge sharing and creation (Chen and Huang, 2007), which lead to high effectiveness for both individuals and organizations (Coleman, 1988; Leana and Pil, 2006; Oh et al., 2006).

The importance of individual social ties on employee performance such as innovation performance and creativity has been underscored in high-tech enterprises (Hu, 2008; Zhou et al., 2009). In these firms, many professionals are heavy users of tacit knowledge, meaning that the knowledge needed by these individuals are not obtained readily from an organization’s formal documents (Liu and Liu, 2008). Relatedly, when they encounter difficult technical or professional problems, they usually first seek assistance and support from their colleagues. Moreover, these employees usually exchange the knowledge and resources to those with high-quality interpersonal relationships colleagues (Bouty, 2000). Consequently, high-quality social ties in high-tech firms is an important factor that employees to integrate resources, exchange information and gain support, which all contribute to their individual performance. Nevertheless, few studies have explored the antecedents of social ties within an organizational context.

### Collaborative HRM and employee intra-organizational social ties

We propose that organizations can implement collaborative HRM practices to enlarge and strengthen employees’ social ties with their colleagues. HRM practices such as selecting job candidates based on their collaborative ability, providing training programs focusing on interpersonal relation building skills, and implementing mentoring systems can improve employees’ abilities to build social ties with their colleagues. HRM practices like teamwork-based performance appraisal and compensation design, promotion based on the ability to work with others can motivate employees to have interaction with more colleagues in a more often and closer manner. Organizations can also provide opportunities for employees to interact with their colleagues by adopting HRM practices such as providing time and resources for internal social activities, designing an interdependent working structure, implementing job rotation. Taken together, when organizations implement collaborative HRM practices, they foster the abilities, motivation, and opportunities necessary for social tie formation on the employees side.

We further argue that collaborative HRM practices influence employees’ intra-organizational social ties through three mechanisms, namely, the assortative, relational and proximity mechanisms (Rivera et al., 2010). First, collaborative HRM help employee develop social ties through an assortative mechanism. HRM practice such as selecting job candidates based on their collaborative abilities, providing training focusing on teamwork skills will increase employees’ common trait of collaborative spirit and abilities. Research has shown that individuals tend to foster close relationships with similar others (Brass et al., 2004). Consequently, through the assortation due to the implementation of HR practices, employees can increase the chances to interact with similar others in the form of high tie numbers, and also increase the likelihood to develop strong social interactions in the form of high tie strength.

Second, collaborative HR practices activate a relational mechanism of network building. Collaborative HR practices facilitates employees’ social tie formation in the form of the tie number. HR practices such as team-based design, job rotations, provide formal opportunities for employees to interact with other colleagues. Informally, training, and company social events also provide more opportunities for employees to socialize. Moreover, the appraisal, rewarding, and promotion practices emphasizing collaborative results further encourage employees to work together. Moreover, collaborative HR practices further promote employees develop high tie strength. This is because, with the opportunities to work together, employees are likely to foster high-quality social tie as a consequence of reciprocation and acquaintance. Past research has documented that individuals tend to develop meaningful social ties in a long run when favors are reciprocated during interactions, and when work with those they have worked with in the past (Guimera et al., 2005).

Third, collaborative HRM practices facilitate employees to develop their social tie through a proximity mechanism, which means that individuals tend to formulate social ties when they are located within a short distance either in terms of physical space or social foci (Rivera et al., 2010). Interdependent work design and information sharing meetings give employees more opportunities to be closely located in the workplace. Sponsoring company social events and informal organizations allows employees to meet together more often in the same physical space and concurrently reduces the social distances among them. Team-oriented appraisal and reward systems also direct employees’ interests toward their common goals and make them feel close with each other socially. Similar to the abovementioned relational mechanism, these opportunities for coordinate and work together can lead to strong social interactions in terms of both tie number and strength.

Based on the above theoretical arguments, we propose the following hypothesis:

*Hypothesis 1a*: *Collaborative HRM is positively related to the number of employees’ intra-organizational social ties*.

*Hypothesis 1b*: *Collaborative HRM is positively related to the strength of employees’ intra-organizational social ties*.

### Climate for cooperation as the mediator

Organizational HR systems can build a strong social context to guide employees’ expectations and behaviors (Ferris et al., 1998; Bowen and Ostroff, 2004; Collins and Smith, 2006). Researchers use the concept of organizational climate to describe such social contexts. Organizational climate can be defined as organizational members’ perception of formal and informal organizational policies, practices, and procedures (Schneider et al., 1998). Lepak et al. (2006, p. 224) argued that, “Organizational climate has been positioned as a key intermediate variable between organizational context and work outcomes. Specifically, organizational practices, policies, and procedures are argued to influence organizational climate, while organizational climate influences employees’ collective attitudes and behaviors, which in turn influence organizational effectiveness.” As a result, we propose that the organizational climate may play a mediating role in the relationship between the collaborative HRM and employees’ behaviors relating to social interactions. Following past research in SHRM, instead of using a global climate concept, we focus on the climate for cooperation to represent the specific focus of strategic focus of the organization (Schneider et al., 1998; Lepak et al., 2006). Collins and Smith (2006) defined a climate for cooperation as the organizational norms that emphasize personal efforts toward group outcomes rather than individual outcomes. Nahapiet and Ghoshal (1998) argued that a climate for cooperation is a norm that significantly influences the exchange process, which opens the channel for other groups to exchange knowledge and determine the motivation for such an exchange. Thus, we consider a climate for cooperation as the collective norm perceived by employees, in which employees are expected and rewarded to communicate and cooperate with others.

Following Lepak et al. (2006), we propose that the positive relationships between the collaborative HRM and employee intra-organizational social ties (tie number, and tie strength) are mediated by the climate for cooperation. Ample research has shown that HRM can create an organizational climate that elicits certain behaviors from employees (Ferris et al., 1998; Collins and Smith, 2006; Lepak et al., 2006). Similarly, collaborative HR practices, such as promotion based on the ability to work with others, training on intra-firm relationship-building and selecting job candidates on the basis of their ability to collaborate in teams, interact with each other, consistently sending clear signals to employees that internal exchange and interactions are expected and rewarded by the organizations, thus contributing to the shared beliefs and perceptions regarding cooperative norms. In other words, when organizations adopt collaborative HR practices, the employees will observe a strong climate for cooperation.

When the norm of cooperation is established, it is natural to expect employees to interact with their colleagues more extensively and more frequently, since cooperation and internal exchange will be expected and rewarded by the organizations. According to the social identity theory, employees’ social and work behaviors should be socialized under the influence of the common values of the organization (Louis, 1980). Expectedly, members within an organization would behave according to the common characteristics of the organization and make their cognition and behavior consistent with other members in the organization. Therefore, the cooperation climate would guide employees to actively communicate and interact with other members of the organization in order to maintain the identity of members of the organization. This will increase employees social tie numbers and strength. On the contrary, if the cooperative climate is weak or nonexistent, employees would observe that the organization has lower requirements on interaction with colleagues, and thus employees’ behavior of building social connections would be drastically reduced (Chen and Huang, 2007).

Moreover, a climate for cooperation also promotes the formation of employee social networks by reducing the sense of competition among the employees. For knowledge workers, knowledge is a source of power and job security (Davenport and Prusak, 1998), therefore, they tend to have reserved attitudes towards sharing knowledge and information with their colleagues, which undermine the sharing and diffusion of knowledge (Reagans and McEvily, 2003; Collins and Smith, 2006). However, a climate for cooperation can reduce the sense of competition among employees by motivating them to focus more on the cooperation than on individual performance (Ingram and Roberts, 2000). Consequently, employees will be encouraged to interact with their colleagues and share what they know. Therefore, we propose the following hypothesis:

*Hypothesis 2a: Climate for cooperation mediates the positive cross-level relationship between collaborative HRM and the number of employees’ intra-organizational social ties*.

*Hypothesis 2b: Climate for cooperation mediates the positive cross-level relationship between collaborative HRM and the strength of employees’ intra-organizational social ties*.

## Materials and methods

### Sample and research procedures

The data for this study were elicited from online questionnaire surveys which were distributed to information technology (IT) companies in *Zhongguancun*, Beijing, China. This survey was conducted with the assistance of the *Beijing Zhongguancun Association of IT Professionals*. Due to the multiplicity of employee groups and subsequent multiplicity of HR systems within a firm (Lepak and Snell, 1999, 2002), our survey only focused on core knowledge employees whose primary duties and responsibilities are mainly related to R&D departments, engineering projects, and related departments. We ensured that the data for collaborative HRM were answered by both line managers and human resource managers, while the data of climate for cooperation and employee social ties were answered by core knowledge employees.

Overall, 126 out of the 213 firms that agreed to participate in the survey returned the questionnaires (59.1% return rate). Consequently, 126 HR managers, 287 line managers, and 548 employees completed the questionnaire. We included only firms that provided data for HR managers, line managers, and at least three core knowledge employees, thus reduced the number of qualified firms to 64, with 5.7 employees (range 3–17), 3.3 line managers (range 1–13) and 1 HR manager per firm on average. The sampled firms were, on average, 11.8 year old (sd = 7.1, min = 2, max = 38), hiring 815 employees (sd = 1,115, min = 44, max = 5,272). Furthermore, among the sampled core knowledge employees, 56.0% were male and 44.0% were female, averaging 27.4 years of age and 2.2 years of work experience at their current respective firms, while 98.0% had either college degrees or higher qualifications.

### Measures

#### Collaborative HRM

The measurement of collaborative HRM concentrates on employees’ abilities, motivation, and opportunities for collabation and network building. These items were adapted from three studies that explicitly use the concept of collaborative HRM (Lepak and Snell, 2002; Youndt and Snell, 2004; Lopez-Cabrales et al., 2009). At the same time, we refer to the research on SHRM with high-tech enterprises (Collins and Smith, 2006) and core knowledge employees (Kang et al., 2007) as the research objects. In order to better adapt to the Chinese context, we also conducted interviews with HR managers in IT companies to supplement the responses derived from the questionnaire survey. Finally, an initial questionnaire of 16 items was formed. We then used two methods to improve validity. On the one hand, we tested the content validity by a professional team composed of 10 management experts. The members of the expert panel responses were scored 1–4 for each item of the scale (1 for no correlation, 2 for weak correlation, 3 for strong correlation, and 4 for strong correlation). For each item, the number of experts with a score of 3 or 4 divided by the total number of experts participating in the evaluation is the content validity index (CVI) of the corresponding item. Likewise, we performed factor analysis on the initial questionnaire. Finally, we eliminated the items with CVI score below 0.8 and streamlined the scale according to the “model modification indices” provided by Mplus 8.3 (Lynn, 1986; Davis, 1992). Ultimately, a questionnaire including 11 items was formed. The measurement items includes the following: Training and development practices aimed at improving employees’ cooperation and social ability; Performance, compensation, and promotion practices aimed at improving employee cooperation and social motivation; Formal work design practices aimed at enhancing opportunities for collaboration and networking among employees. A five-point Likert scale was used to measure the magnitude of all items.

Since this paper uses the scale of collaborative HRM for variable measurement, it is necessary to test the reliability and validity of this construct. Therefore, a total of 268 valid questionnaires were collected from HR managers and line managers. Exploratory factor analysis (KMO = 0.92，*p* < 0.001) was used to extract three factors by principal component maximum variance rotation method. The results showed that the cumulative variance contribution rate of the three factors was 73.49%, and the factor loading of each item was greater than 0.6. As shown in Table 1, the rotated component matrix shows a clear three-factor structure. The fitting index of collaborative HRM three-factor model (χ^2^/df = 2.482，RMSEA = 0.075，CFI = 0.966，TLI = 0.954，SRMR = 0.033) is shown in Table 2. Findings from Table 2 reveal that the fitting index of the three-factor model is significantly better than that of the two-factor model and the single-factor model. Therefore, consistent with theoretical expectations, collaborative HRM is a three-dimensional construct, which is reflected in improving employees’ social ability through training and development practices, stimulating employees’ social motivation through assessment, compensation and promotion practices, and providing social opportunities through formal job design. The alpha value of each dimension is greater than 0.8. Moreover, the alpha for the scale was 0.92.

**Table 1:** The results of exploratory factor analyses for collaborative HRM.

Factor	Items	Loading			Cronbach’s α
CHRM-A	Providing training focusing on team-building and teamwork skills	0.76			0.86
	Providing career path opportunities for employees to move across multiple functional areas of the company	0.70			
	Sponsoring company social events for employees to become acquainted with one another	0.75			
	Conducting information-sharing meetings for employees to know more internal information about the enterprise	0.78			
CHRM-M	Promotion based on abilities to work with others		0.68		0.87
	Utilizing group-based incentives		0.79		
	Merit-based raises based on teamwork skills and team orientation		0.82		
	Monetary rewards based on the outcomes of interdependent tasks		0.82		
CHRM-O	Most of the work is performed through teamwork			0.81	0.84
	Most of work is interdependent, rather than independent of each other			0.78	
	Building cross-functional teams to complete the work			0.72	

Extraction method, principal component analysis; Rotation method, varimax with Kaiser normalization; CHRM-A, the practices of ability dimension in collaborative HRM; CHRM-M, the practices of motivation dimension in collaborative HRM; CHRM-O, the practices of opportunity dimension in collaborative HRM.

**Table 2:** The results of confirmatory factor analyses for collaborative HRM.

Models	χ^2^	df	χ^2^/df	RMSEA	CFI	TLI	SRMR
Three-factor model	101.761	41	2.482	0.075	0.966	0.954	0.033
Two-factor model^a^	175.153	43	4.073	0.107	0.926	0.905	0.044
Two-factor model^b^	250.546	43	5.827	0.134	0.883	0.851	0.055
Two-factor model^c^	262.010	43	6.093	0.138	0.877	0.842	0.056
One-factor model	332.848	44	7.565	0.157	0.837	0.797	0.063

^a^The ability dimension and the opportunity dimension were combined in two-factor model. ^b^The ability dimension and the motivation dimension were combined in two-factor model. ^c^The motivation dimension and the opportunity dimension were combined in two-factor model.

Since there are criticisms regarding the use of a single response to measure company HRM practices, we then constructed the collaborative HRM index by averaging the index score of the HR manager and that of the line managers. The ICC(1) was 0.09, while the average of the Rwg_(j)_ for the collaborative HRM index was 0.9, indicating that the aggregation was justified (Bliese, 2000).

#### Climate for cooperation

Climate for cooperation was measured by a 4-item scale adapted from Chatman and Flynn (2001). Items included “it is important to maintain harmony among employees within the organization,” “employees in this organization are willing to sacrifice their self-interest for the benefit of the organization,” “There is a high degree of cooperation among employees in this company,” and “There is a high sense of sharing among employees in this company.” The Cronbach’s alpha for the scale is 0.73. We then averaged the responses of employees in each company as core knowledge employees’ perceived organizational climate for cooperation. The mean Rwg_(j)_ was 0.85 and the ICC(1) was 0.16, which can justify such aggregation to the organization-level in this study (Bliese, 2000).

#### Employee intra-organizational social ties

Following Collins and Clark (2003) and Pil and Leana (2009), employee intra-organizational social ties were measured by tie number and tie strength. The tie number was measured by the question “How many colleagues within the company do you regularly discuss expertise or exchange technical information with?.” The response options range from 1 to 7 (1 represents “0,” 2 represents “1,” 3 represents “2,” 4 represents “3,” 5 represents “4,” 6 represents “5,” and 7 represents “6 or above”). The tie strength was measured as the average of frequency and closeness (Collins and Clark, 2003; Pil and Leana, 2009). Frequency was measured as the number of times that the employees spoke to their peers to gain expertise and related information during the last month (1 = “0–5 time(s),” 2 = “6–10 times,” 3 = “11-15times,” 4 = “16–20 times” and 5 = “more than 21 times”). Closeness was measured by a one-item 5-point scale “In general, how closely do you feel you discuss expertise or exchange technical information with your colleagues?” (1 = not at all close; 5 = very close).

#### Control variables

At the individual level, we controlled for the employees’ gender, age, tenure, and education to avoid experience-related prejudice and gender effects (Snape and Redman, 2010). We also controlled for the following organizational variables: firm age (years since the legal establishment), firm size (natural logarithm of incumbent full-time employees) as previous studies suggested (Takeuchi et al., 2007).

#### Confirmatory factor analysis and Harman’s single-factor test

We conducted a series of confirmatory factor analyses (CFA) to test the measurement model specifying collaborative HRM, climate for cooperation, and employees’ intra-organizational social ties as separate factors. Table 3 presents the CFA results. As shown, the hypothesized 3-factor model (χ^2^/df = 0.659，RMSEA = 0.000，CFI = 1.000，TLI = 1.048，SRMR = 0.022) fits the data better than the 2-factor model^a^ (χ^2^/df = 2.700，RMSEA = 0.069, CFI = 0.835, TLI = 0.759, SRMR = 0.097), 2-factor model^b^ (χ^2^/df = 3.501, RMSEA = 0.083, CFI = 0.729, TLI = 0.646, SRMR = 0.127), and the 1-factor model (χ^2^/df = 6.664, RMSEA = 0.125, CFI = 0.340, TLI = 0.198, SRMR = 0.180). Therefore, these results suggest that our measures’ exhibit discriminant validity among these constructs, since each measure is conceptually distinct.

**Table 3:** The results of confirmatory factor analyses for measurement model.

Models	χ^2^	df	χ^2^/df	RMSEA	CFI	TLI	SRMR
Three-factor model	21.101	32	0.659	0.000	1.000	1.048	0.022
Two-factor model^a^	94.487	35	2.700	0.069	0.835	0.759	0.097
Two-factor model^b^	136.553	39	3.501	0.083	0.729	0.646	0.127
One-factor model	279.868	42	6.664	0.125	0.340	0.198	0.180

^a^Climate for cooperation and social ties were combined in a two-factor model. ^b^Collaborative HRM and climate for cooperation were combined in a two-factor model.

We next ran Harman’s single-factor test using all the items at the individual level to examine the potential common method variance problem (Podsakoff et al., 2003). The result showed that the single factor accounted for approximately 27.65% of variance, which is less than the threshold (i.e., 50%). Based on this result, we conclude that common method variance is not a serious issue in our dataset.

#### Analytical approach

We use SPSS 23.0 to perform the descriptive statistics and correlations, and Mplus 8.3 to test the cross-level mediation-upper mediator model.

First, we applied Kurtosis and Skewness to check the data normality following Muthén and Kaplan (1985, 1992) and Ferrando and Anguiano-Carrasco (2010), who recommend coefficients of skewness and kurtosis in a range of-1, 1. All the variables in our hypothesized model were normally distributed with Skewness ranging between-1 and 1 (Collaborative HRM = -0.033; Climate for cooperation = −0.011; Tie number = 0.115; Tie strength = 0.232) and Kurtosis ranging between-1 and 1 (Collaborative HRM = 0.025; Climate for cooperation = −0.013; Tie number = −0.804; Tie strength = −0.505). To justify that the data is appropriate for analyzing two-level model, we began with a null model to calculate the intra-class correlation coefficient (ICC). The results provided an ICC(1) = 0.112 for employee tie number and an ICC(1) = 0.073 for employee tie strength, both of which are higher than the 0.059 recommended by Cohen (1988). The method used in the estimation of statistical outputs is robust maximum likelihood estimation, which has the advantage as less sensitive to the number of samples, while the commonly used maximum likelihood estimation is more sensitive to the number of samples and missing data.

To test the significance of the indirect effects, A Monte Carlo simulation with 20,000 replications was conducted to test the 95% bias-corrected confidence interval (CI), using the web estimator provided by Selig and Preacher (2008). This method has been suggested to determine indirect effects in multilevel models (Preacher and Selig, 2012). If the 95% CI does not include zero, we can conclude that the indirect effect is significant. Based on Hu and Bentler (1999) recommendations, means of the root-mean-square error of approximation (RMSEA), the Tucker–Lewis Index (TLI), and the comparative fit index (CFI), and the standardized root mean square residual (SRMR) were employed to assess model fit. The following cut-off values were used: RMSEA values below 0.08, TLI values higher than 0.8, CFI values higher than 0.8, and SRMR values below 0.08.

## Results

### Descriptive statistics and correlations

The mean, standard deviation, and correlation coefficient matrix of the variables are shown in Table 4.

**Table 4:** Descriptive statistics, means, standard deviations, and correlations.

Variables	*M*	*SD*	1	2	3	4	5	6
Individual level (*N* = 363)								
1. Age	27.43	4.79						
2. Gender	1.45	0.49	0.79					
3. Edu	2.89	0.58	0.27	0.49				
4. Work tenure	2.20	1.93	0.49**	0.77	−0.13*			
5. CC	3.66	0.74	−0.76	0.88	−0.07	0.01		
6. TN	4.66	1.54	−0.18	−0.02	0.07	−0.03	0.31**	
7. TS	2.96	0.94	−0.13*	−0.09	0.01	−0.06	0.26**	0.49**
Firm level (*N* = 64)								
1. Firm age	11.84	7.12						
2. Firm size	5.94	1.29	0.41**					
3. CHRM	3.40	0.47	−0.14	−0.04				
4. CC	3.67	0.44	−0.31	0.12	0.31*			

CHRM, collaborative HRM; CC, climate for cooperation; TN, tie number; TS, tie strength. *p < 0.05, **p < 0.01.

### Hypothesis tests

The results of hypothesis test are shown in Table 5. In Model 1, we estimated the independent variable collaborative HRM’s direct effect on the dependent variable (i.e., tie number and tie strength), with individual-level control variables and organizational-level control variables. The results show collaborative HRM has a positive effect on tie number (*b* = 0.574, *p* < 0.05) and tie strength (*b* = 0.406, *p* < 0.01). Therefore, Hypothesis 1 is supported.

**Table 5:** The model results.

Variables	Model 1		Model 2	Model 3		Model 4		
	TN	TS	CC	TN	TS	CC	TN	TS
Intercept	1.679	2.105**	2.350***	0.208	2.227***	5.662***	−0.454	1.43*
Individual level								
Age	0.004	−0.018		−0.003	−0.024		0.001	−0.019
Gender	−0.097	−0.187		−0.072	−0.166		−0.092	−0.189
edu	0.159	0.033		0.137	0.007		0.144	0.027
Work tenure	−0.011	0.006		−0.008	0.020		−0.008	0.007
Organizational level								
Firm age	−0.003	0.004	0.000	−0.003	0.002	−0.049	−0.001	0.005
Firm size	0.108	0.011	0.025	0.078	−0.003	0.150	0.082	0.003
CHRM	0.574*	0.406**	0.338***			0.307*	0.242	0.296*
CC				1.048***	0.419**		0.956***	0.309*
R^2^	0.450	0.567	0.135	0.884	0.590	0.114	0.896	0.809

*n* = 363 for individual-level variables. *n* = 64 for organization-level variables. CHRM, collaborative HRM; CC, climate for cooperation; TN, tie number; TS, tie strength. The coefficient is non-standard coefficient; R^2^ calculated by Mplus 8.3 according to the normalization coefficient. *p < 0.05, **p < 0.01, ***p < 0.001.

In Model 2, we estimated the direct effect of the independent variable (i.e., collaborative HRM) on the mediating variable (i.e., climate for cooperation). The regression results show that a collaborative HRM system is positively related to the climate for cooperation (*b* = 0.338, *p* < 0.001).

In Model 3, we examined the effect of the mediating variable (i.e., climate for cooperation) on the dependent variable (i.e., tie number and tie strength). Our results indicate that the climate for cooperation was significantly associated with tie number (*b* = 1.048, *p* < 0.001) and tie strength (*b* = 0.419, *p* < 0.01).

**Figure 2:**
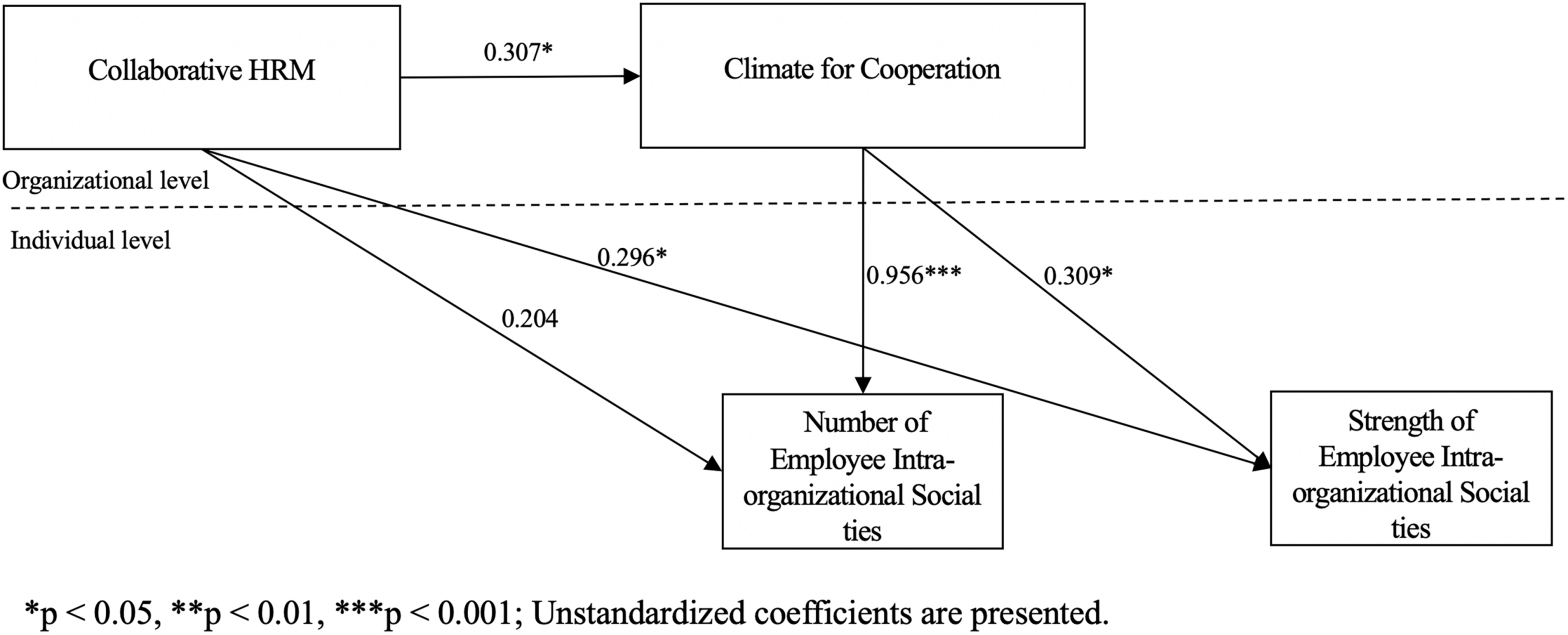
Results of multilevel mediation analysis.

In Model 4, we examined the mediating role of climate for cooperation. When we put the independent variable and the mediating variable together in the regression model, the results show that the mediating variable (i.e., climate for cooperation) has a significant positive relationship with tie number (*b* = 0.956, *p* < 0.001) and tie strength (*b* = 0.309, *p* < 0.05), whereas the relationship between collaborative HRM and tie number decreased from 0.574 (*p* < 0.05) to 0.242 (*p* > 0.05), and the relationship between collaborative HRM and tie strength decreased from 0.406 (*p* < 0.01) to 0.296 (*p* < 0.05). Results showed that the indirect effects between collaborative HRM and tie number (indirect effect = 0.271, 95%CI = [0.0393, 0.5596]) and tie strength (indirect effect = 0.087, 95%CI = [0.0041, 0.2121]) through climate for cooperation were significant. The overall results of the proposed model indicated an acceptable level of model fit (RMSEA = 0.000, TLI = 1.000, CFI = 1.000, SRMR within = 0.002, SRMR between = 0.023). Consequently, we concluded that a climate for cooperation mediates the relationship between collaborative HRM, and the number and strength of employees’ intra-organizational social ties. Thus Hypothesis 2 is supported (see Figure 2).

### Additional tests

In previous studies, the collaborative HRM construct has been used to emphasize the dimensions of ability and motivation (Lopez-Cabrales et al., 2009), however, this study integrates the opportunity dimension into this construct. Hence, we compared both the total and indirect effects of collaborative HRM as proposed in this study, and the traditional collaborative HRM model which includes only the ability and motivation dimensions. The ensuing comparison of these total effects are shown in Table 6, and the comparison of indirect effects to the adjusted model are shown in Table 7. The results show that the effectiveness of collaborative HRM with three dimensions of ability, motivation and opportunity is better than that with only two dimensions of ability and motivation.

**Table 6:** Comparison of total effects between different dimensions in CHRM.

Variables	R^2^			Total effect		
	TN	TS	CC	TN	TS	CC
CHRM-A	0.303	0.400	0.093	0.389*	0.294**	0.238***
CHRM-M	0.355	0.526	0.124 0.122	0.405	0.325**	0.270***
CHRM-AM	0.363	0.527		0.452*	0.352**	0.288***
CHRM-AMO	0.450	0.567	0.135	0.574*	0.406**	0.338***

CHRM, collaborative HRM; CHRM-A, the practices of ability dimension in CHRM; CHRM-M, the practices of motivation dimension in CHRM; CHRM-AM, the combination of the practice of ability dimension and the practice of motivation dimension in CHRM; CC, climate for cooperation; TN, tie number; TS, tie strength. the coefficient is non-standard coefficient; R^2^ calculated by Mplus 8.3 according to the normalization coefficient. *p < 0.05, **p < 0.01, ***p < 0.001.

**Table 7:** Comparison of indirect effects between different dimensions in CHRM.

Variables	R^2^			Indirect effect		90% percent	
	TN	TS	CC	TN	TS	TN	TS
CHRM-A	0.891	0.762	0.098	0.225 (0.048)	0.078 (0.086)	[0.038, 0.413]	[0.003, 0.153]
CHRM-M	0.878	0.775	0.083 0.099	0.192 (0.081)	0.063 (0.136)	[0.011, 0.372]	[−0.006, 0.131]
CHRM-AM	0.885	0.797		0.232 (0.052)	0.076 (0.101)	[0.036, 0.429]	[0.000, 0.152]
CHRM-AMO	0.896	0.809	0.114	0.271 (0.035)	0.087 (0.091)	[0.059, 0.482]	[0.002, 0.172]

CHRM, collaborative HRM; CHRM-A, the practices of ability dimension in CHRM; CHRM-M, the practices of motivation dimension in CHRM; CHRM-AM, the combination of the practice of ability dimension and motivation dimension in CHRM; TN, tie number; TS, tie strength. The coefficient is non-standard coefficient; R^2^ calculated by Mplus 8.3 according to the normalization coefficient. () = value of *p* appear in parentheses.

## Discussion

### Theoretical implications

The objective of this study is to examine the cross-level relationship between collaborative HRM and employee’s intra-organizational social ties, as well as the mediating role of the climate for cooperation in this relationship. This study has several implications for both theory and practice.

First, our study revealed that organizational level collaborative HRM and climate for cooperation positively influence employees’ intra-organizational social ties, which contributes to the social network literature since few studies have investigated how organizational contexts affect employee social networks in a cross-level way. Especially in high-tech enterprises, social ties within an organization play a key role in knowledge transfer and sharing (Hu, 2008; Zhou et al., 2009), thus organizations need to guide the cooperation and exchange among employees through well-articulated policies such as HRM (Patel et al., 2013; Garaus et al., 2016). Although this phenomenon has received increasingly attention from scholars in recent years (Methot et al., 2018; Soltis et al., 2018), the empirical research about cross-level effects of organizational context factor on employees’ social ties is still rare. Thus, this paper expands the antecedent variables in the field of social networks.

Second, the finding highlights the importance of collaborative HRM practices in knowledge intensive firms, and reveals an important path for collaborative HRM to exert its effectiveness by incorporating the organizational climate. Since the social interactions and social networks play important roles in knowledge sharing and creation (Reagans and McEvily, 2003; Hansen et al., 2005; Chen and Huang, 2007), HRM in organizations need promote the social relationships among employees. However, a large number of previous SHRM studies have focused on high-performance work systems, with limited investigations of other forms of HRM. Our study found that collaborative HRM practices have a significant cross-level influence on employee intra-organizational social ties, which enrich our understanding of the collaborative HRM and call for a deeper and more theoretical interpretation of SHRM research in the knowledge economy. Besides, our study found that organizational climate for cooperation play a mediating role, which is consistent with the findings of previous studies that have taken the social climate as a mediation variable of SHRM (Prieto and Santana, 2012; Cooper et al., 2019).

Third, using the AMO model, our study improves the construct and measurement of collaborative HRM, thus expanding the configuration of SHRM. In the SHRM field, different configurations and specific-target orientations are becoming more and more important (Shen and Benson, 2014; Chuang et al., 2016; Lee et al., 2019). Collaborative HRM is an important configuration of SHRM, which is characterized by investing in interpersonal relationships. However, in the process of developing this construct, previous studies did not adequately use relevant theories to define and examine the measure structure of this important construct, and did not conduct a CFA test. Compared with the previous research (Lopez-Cabrales et al., 2009), we proposed a clearer ability-motivation-opportunity three-dimensional structure of collaborative HRM. Through factor analysis, hypothesis testing and additional testing, we verified the dimensions of the construct, and its effect on employees’ social ties, thus enriching our understanding of collaborative HRM, as well as expanding the configuration of SHRM from the perspective of social network.

### Practical implications

This study has important implications for management practices. This is because the competitive advantage of high-tech firms are derived from knowledge sharing and creation. Interestingly, collaborative HRM can create a cooperative climate where employees collaborate with their colleagues, and also expand their social ties within an organization, consequently promoting knowledge sharing and creation. This implies that for IT firms, it is risky to simply invest in employees’ human capitals. Given the fact that there is fierce talent competition and a high rate of turnover in the IT industry, we suggest that this may impose an organization to a high investment risk. Comparatively, rather than investing in individual employees, collaborative HRM emphasizes investment in employees’ social networks, which are embedded within organizations and cannot be easily dissolved when a focal employee leaves a company. Thus, it provides high-tech firms with both a useful tool to promote knowledge exchange, and an opportunity to enhance the human capital skillsets of their employees. Therefore, designing and implementing collaborative HRM systems in an organization is essential for IT firms, in order to gain competitive advantage in a knowledge-driven economy.

### Limitations and future directions

Our study has the following limitations. First, because studies on collaborative HRM are relatively few and sketchy, the items in our questionnaire may not completely cover every aspect of a collaborative HRM system. Future studies should therefore explore the composition of a collaborative HRM system in greater details. Second, because the goal of this study is to investigate how organizational factors influence employee social ties, we measured the collaborative HRM at the organizational level using the managers reports rather than employees’ perceptions of HR practices. However, previous studies have revealed that employees’ perceptions of HR practices can significantly influence their work attitudes and behaviors, and may also mediate the relationships between organization intended HR practices and employee outcomes (Wang et al., 2020). Thus future research can incorporate employee percpetions of HR practices by investigating how organizations can improve employees’ perceptions of a collaborative HRM system, and the conditions under which employees’ perceptions of collaborative HR practices can mediate the relationships between organizational level collaborative HRM and individual level social ties. Third, one assumption of this study is that employee social ties are positively related to knowledge sharing and creation, and ultimately employee performance. Although many empirical studies have provided the related evidence, our empirical investigation did not include knowledge sharing and creation, and employee performance. Future studies may examine the causal chain linking collaborative HRM and employee performance as a complete model. Fourth, though we had several characteristic variables of employees and organizations as the controls, we did not consider the influences of personality characteristics on individual social networks. Past research has demonstrated that personal characterstics such as proactive personality (Thompson, 2005), self-monitors (Sasovova et al., 2010) and cooperative orientation (Chen X. P. et al., 2011) can influence personal social network formation. For future research, on the one hand, these personality traits can be included as control variables, so as to study the impacts of collaborative HRM on employees’ social ties in a more accurate way. On the other hand, these personality traits may be regarded as boundary condition. For examples, employees with high proactive personality or cooperative orientation may hold more intrinsic motivations to expand their social interactions, which may weaken the impacts of collaborative HRM on employees’ social ties. Thus, future research can include employee personality variables in the research model to better understand the relationships between HR practices and social ties from a more employee-focused perspective.

## Conclusion

In closing, the primary goal of this study was to examine how the organizational-level collaborative HRM systems affect individual-level intra-organizational social ties. Based on the analysis of the dataset comprising 363 knowledge employees from 64 high-tech firms in China, the results showed that collaborative HRM had significant positive effects on the number and strength of individual-level intra-organizational social ties, and the climate for cooperation mediates these relationships. Our study not only advances the knowledge concerning how organizational contexts affect employee social networks in a cross-level way, but also reveals an important path for collaborative HRM to exert its influences by considering the organizational climate.

## Data availability statement

The raw data supporting the conclusions of this article will be made available by the authors, without undue reservation.

## Ethics statement

The studies involving human participants were reviewed and approved by Academic Ethics Committee of Renmin University of China. The patients/participants provided their written informed consent to participate in this study. Written informed consent was obtained from the individual(s) for the publication of any potentially identifiable images or data included in this article.

## Author contributions

ZS and XL: conceptualization, methodology, and writing-original draft preparation. MZ and XL: data curation, software, writing, reviewing, and editing. MZ, YY, and WS: visualization, investigation, supervision, and validation. All authors contributed to the article and approved the submitted version.

## Conflict of interest

The authors declare that the research was conducted in the absence of any commercial or financial relationships that could be construed as a potential conflict of interest.

## Publisher’s note

All claims expressed in this article are solely those of the authors and do not necessarily represent those of their affiliated organizations, or those of the publisher, the editors and the reviewers. Any product that may be evaluated in this article, or claim that may be made by its manufacturer, is not guaranteed or endorsed by the publisher.
